# Our New York Letter

**Published:** 1891-10

**Authors:** Wm. L. Russell

**Affiliations:** 151 East Fiftieth Street; New York


### OUR NEW YORK LETTER.

NEW YORK, September 19, 1891.

Medical matters have been peculiarly stagnant in New York this summer. There have been no medical meetings, no startling discoveries, and even the customary summer diseases have not been so prevalent, in spite of the fact that several miles of streets have been constantly dug up by the railroad and gas companies.

The house to house visitation in the tenement house districts, by physicians of the Health Department, has been prosecuted as usual during the hot season. This work has already borne good fruit here, and is worthy of imitation by other large cities. The corps consists of fifty physicians, to each one of whom is assigned a district. Within this district, it is his duty to visit each family living in a tenement house and ascertain the sanitary condition of the premises and of the different apartments, the habits of the people in reference to sanitary matters and the care of their children, and in cases in which the family is too poor to pay a physician to attend any who are sick. The importance of such work in this city can only be thoroughly appreciated by those familiar with the crowded condition of some of the districts, and the uncleanly habits of the people. The recent influx of Russian Jews has crowded parts of the city to a point beyond anything in any civilized city in the world. These people and the Italians are uncleanly to a degree. Sanitary regulations are of little avail, and even the constant personal supervision of a health officer hardly prevents their quarters from being pest-breeding nests. Much good in the aggregate is, however, accomplished by the summer corps. Many children are saved by the prompt treatment of what the mother regards as an insignificant diarrhœa or cough, and the continued distribution of circulars giving instructions concerning infant feeding has brought a more intelligent understanding of this subject among the poor and ignorant. The result of this is beginning to be noticeable. The usual plan of treatment of moderate diarrhœa is to correct errors in the feeding, which I may say is found in the large majority of cases, and give a powder containing one grain of pepsin, two grains of bismuth and two of aromatic chalk powder every two or three hours. As an adjunct to this treatment sea air is generally added. This is obtained by means of the Floating Hospital of St. John's Guild. This hospital consists of a large barge which has been fitted up for the purpose. It carries physicians and nurses, and every means is provided for the care of the sick. The barge is towed down the bay in the morning and remains there all day, returning in the evening. As many as 1,600 mothers and children are taken on a single trip.

Another duty of the visiting physician, and a more difficult one, is to instruct the people in sanitary matters. Advice regarding the cleaning and ventilating of rooms, the disposal of kitchen refuse, the airing of bedding, etc., is offered, and in some cases its practical adoption must be enforced at the moment. This is the most difficult matter to contend with in all the sanitary reforms. It is hard to alter the habits of the individual. The problem must be solved by the force of example, the distribution of literature bearing on the subject, and the instruction of children in the public schools. The natural advantages of New York ought to render it one of the most healthful cities in the world, and yet the death rate is as high as that of any large city in the temperate zone.

### IMPETIGO CONTAGIOSA COMPLICATING VACCINATION.

A skin eruption unusual in its severity, if not in its nature, has been seen attending vaccination in a few cases in this city. One of them chanced to come under my own observation, and a description of it will suffice for those of the others of which I have heard.

It was in a female child of five years of age. She was vaccinated by a health officer in June, and nothing unusual occurred until the pock was fully developed. Then a few blisters appeared in the neighborhood of the pock, which were soon followed by others on other parts of the arm. Then the other arm became affected, and eventually every part of the body, including the face and scalp, was the seat of the eruption in some stage. The vaccination ran the usual course. The patient was not seen by me until five weeks after being vaccinated. There was then nothing to be seen at the seat of vaccination except a scar. The limbs and body of the child, however, presented a striking appearance from the presence of a wide-spread eruption distributed in pronounced outlines. The groupings were principally, although by no means wholly, confined to the flexor surfaces of the arms, the backs of the legs, the inside of the thighs, the genitals and buttocks ; a broad girdle encircled the neck, while the chest and abdomen were entirely free. On close examination the eruption was seen to be bullous in character, the lesions varying in size from two pins' heads to a lima bean, most of them being about as large as a green pea. Where the more pronounced configurations were, the surface was generally reddened and irregularly crusted. On this reddened surface where lesions had just recovered, new blebs were developing, some small and filled with clear fluid, others larger and more opaque. The lesions were usually discrete, but in some instances several had run together, a crusted mass resulting. When this was removed the surface was found excoriated but not ulcerating.

Dr. Geo. T. Elliott, the well-known dermatologist, who saw the case in consultation, drew my attention to the tendency of these patches to spread at the periphery in a serpiginous manner, thus causing the map-like configuration which made the child such a striking picture. Discrete bullæ were also found in the midst of an area of sound skin. They stood out like beads, there being not even a red line of inflammation around them. When these disappeared, which they did by drying into a crust, after rupturing, a red mark was left. The eruption itched fearfully, and the child's health was reduced from the discomfort and broken rest. The case was regarded as one of aggravated impetigo contagiosa, beginning with the vaccination, and spreading principally by anti-inoculation by means of scratching. Free lathering with ichthyol soap was advised by Dr. Elliott, a plan of treatment which relieved the itching immediately and was soon attended by marked improvement. The new lesions became fewer in number and smaller in size, and the child's general health improved very much.

After two weeks a relapse occurred, presumably because of the very hot weather then prevailing. An occasional bath in solution of bichloride of mercury, 1 to 10,000, was then added to the treatment, and ichthyol was used in carron oil instead of soap. The result was not satisfactory, and the use of soap was renewed. About four weeks ago, when again improving, the child was attacked by scarlet fever. The germs of the two diseases appeared to agree amicably, for both eruptions throve equally well. The impetigo lesions were slightly modified, however, in that they were more inclined to become milky and were surrounded by a narrow zone of inflammation.

At present writing the eruption is still present, but is improving under the soap treatment. It is most persistent about the genitals and internal surface of the thighs, where new lesions crop out in clusters in spite of treatment.

The histories of two other cases which have been related to me corresponded essentially with the above. The eruption persisted for four or five months, but eventually disappeared.

151 *East Fiftieth Street.* WM. L. RUSSELL.

---

The Mississippi Valley Medical Association will convene in its seventeenth annual session, St. Louis, October 14, 15 and 16, 1891. The officers are : President, Dr. C. H. Hughes, St. Louis; Secretary, Dr. E. S. McKee, Cincinnati; Chairman Committee of Arrangements, Dr. I. N. Love, St. Louis.

